# Moving toward integrating care in France: factors related to local managers

**Published:** 2012-09-04

**Authors:** Dominique Somme, Matthieu de Stampa, Catherine Périsset, Olivier Dupont, Nadia Arnaout, Joël Ankri, Olivier Saint-Jean

**Affiliations:** Assistance Publique Hôpitaux de Paris, HEGP, Paris, France; Laboratoire Santé Environnement Vieillissement EA 2506, Université de Versailles Saint Quentin, France; Assistance Publique Hôpitaux de Paris, Hôpital Sainte Perrine, Paris, France; Laboratoire Santé Environnement Vieillissement EA 2506, Université de Versailles Saint Quentin, France; Equipe de pilotage nationale, Caisse Nationale de Solidarité pour l’Autonomie, Paris, France; Equipe de pilotage nationale, Caisse Nationale de Solidarité pour l’Autonomie, Paris, France; Equipe de pilotage nationale, Caisse Nationale de Solidarité pour l’Autonomie, Paris, France; Assistance Publique Hôpitaux de Paris, Hôpital Sainte Perrine, Paris, France. Laboratoire Santé Environnement Vieillissement EA 2506, Université de Versailles Saint Quentin, France; Assistance Publique Hôpitaux de Paris, HEGP, Université Paris Descartes, Paris, France

**Keywords:** local management, integrated services delivery, qualitative analysis

## Abstract

**Purpose:**

The 2008–2012 French Alzheimer plan aims to implement an integrated services delivery (ISD) called “Homes for Integration and Autonomy for Alzheimer patients”. Alzheimer disease is taken as a model to implement ISD in the whole system. All pilot projects were driven by a ‘local pilot’ fully dedicated to the ISD implementation.

**Methods:**

We aimed to analyze factors related to the ‘local pilot’ in regard of the implementation of the national public health policy. We conducted interviews of all ‘local pilots’ (n=19) in 2010, their project’s structure managers (n=19) and case managers (17 focus groups). We crossed our results with the external follow-up by the national project team.

**Results:**

Main influencing factors of the ISD implementation were: 1) professional autonomy of the pilot; 2) good positioning of the project’s structure manager; 3) project’s management competency of the pilot; 4) support query by the pilot from the national project team; 5) links between case managers and pilot; and 6) the articulation modality of eventual multiple pilots or managers.

**Discussion and conclusions:**

It was previously shown that it is crucial for ISD implementation to have a dedicated professional for this task. The French national Alzheimer plan gave us an opportunity to analyze factors related to the modality of doing this task.

Powerpoint presentation available at http://www.integratedcare.org at congresses – San Marino – programme.

